# Induction of rapid and selective cell necrosis in *Drosophila* using *Bacillus thuringiensis* Cry toxin and its silkworm receptor

**DOI:** 10.1186/s12915-015-0160-2

**Published:** 2015-07-08

**Authors:** Fumiaki Obata, Shiho Tanaka, Soshiro Kashio, Hidenobu Tsujimura, Ryoichi Sato, Masayuki Miura

**Affiliations:** Department of Genetics, Graduate School of Pharmaceutical Sciences, The University of Tokyo, 7-3-1 Hongo, Bunkyo-ku, Tokyo 113-0033 Japan; Graduate School of Bio-Applications and Systems Engineering, Tokyo University of Agriculture and Technology, 2-24-16 Naka-cho, Koganei-shi, Tokyo 184-8588 Japan; Developmental Biology, Tokyo University of Agriculture and Technology, 3-5-8 Saiwai-cho, Fuchu-shi, Tokyo 183-8509 Japan; CREST, Japan Agency for Medical Research and Development, 20F Yomiuri Shimbun Bldg. 1-7-1 Otemachi, Chiyoda-ku, Tokyo 100-0004 Japan

**Keywords:** Bacillus thuringiensis, Bombyx mori, Cry toxin, Drosophila melanogaster, Genetic ablation, Necrosis

## Abstract

**Background:**

Genetic ablation of target cells is a powerful tool to study the origins and functions of cells, tissue regeneration, or pathophysiology in a human disease model *in vivo*. Several methods for selective cell ablation by inducing apoptosis have been established, using exogenous toxins or endogenous proapoptotic genes. However, their application is limited to cells with intact apoptotic machinery.

**Results:**

Herein, we established a method for inducing rapid and selective cell necrosis by the pore-forming bacterial toxin Cry1Aa, which is specifically active in cells expressing the Cry1Aa receptor (CryR) derived from the silkworm *Bombyx mori*. We demonstrated that overexpressing *CryR* in *Drosophila melanogaster* tissues induced rapid cell death of CryR-expressing cells only, in the presence of Cry1Aa toxin. Cry/CryR system was effective against both proliferating cells in imaginal discs and polyploid postmitotic cells in the fat body. Live imaging analysis of cell ablation revealed swelling and subsequent osmotic lysis of CryR-positive cells after 30 min of incubation with Cry1Aa toxin. Osmotic cell lysis was still triggered when apoptosis, JNK activation, or autophagy was inhibited, suggesting that Cry1Aa-induced necrotic cell death occurred independently of these cellular signaling pathways. Injection of Cry1Aa into the body cavity resulted in specific ablation of CryR-expressing cells, indicating the usefulness of this method for *in vivo* cell ablation.

**Conclusions:**

With Cry toxins from *Bacillus thuringiensis*, we developed a novel method for genetic induction of cell necrosis. Our system provides a “proteinous drill” for killing target cells through physical injury of the cell membrane, which can potentially be used to ablate any cell type in any organisms, even those that are resistant to apoptosis or JNK-dependent programmed cell death.

**Electronic supplementary material:**

The online version of this article (doi:10.1186/s12915-015-0160-2) contains supplementary material, which is available to authorized users.

## Background

Cell ablation followed by phenotypic analysis is an effective method for investigating the function and origin of cells, tissue regeneration, or pathophysiology in a model of human diseases. In contrast to surgical, laser, or pharmacological ablation, genetic ablation is a reproducible, versatile, and technically accessible method for inducing death of target cells *in vivo*. Various genetic ablation methods have been developed. For example, direct induction of apoptosis by expressing modified caspase-8 [1, 2], caspase-3 [3], or caspase-9 [4] under cell type-specific promoters is well established. Exogenous toxins such as the Diphtheria toxin (DT) A subunit have also been used to induce cell death by inhibiting protein synthesis [5, 6]. Conditional expression of the DT receptor allows specific ablation of DT receptor-expressing cells by DT injection [7, 8]. Enzyme-triggered conversion of prodrugs to cytotoxic compounds is another possible strategy [9, 10]. Although the mechanisms of cell death are different, most of these manipulations stimulate the genetically programmed process of apoptosis in the host [11].

In *Drosophila melanogaster*, several genetic ablation methods using DT A, Ricin A, or pro-apoptotic molecules such as Reaper, Hid, Grim (RHG) motif proteins have been established [12]. Targeted cell ablation is usually achieved by combining cell type-specific Gal4 drivers with cell death-inducing genes downstream of the upstream activation sequence (UAS). However, these methods are again predominantly dependent on the apoptotic machinery of target cells. Apoptosis is a time- and energy-consuming process and sometimes results in incomplete cell ablation. Unsuccessful induction of cell death can trigger diverse cellular processes due to mild activation of caspases, confusing the outcome of ablation experiments [13]. Moreover, not all types of cells are susceptible to apoptotic stimuli, as either loss or gain of function of pro- or anti-apoptotic signaling components prevents cells from undergoing apoptosis [14–16]. There is a need for a genetic technique that induces cell death in a more direct physical manner, and can therefore be applied to a wider range of cell types.

Cry toxins are proteinous insecticidal toxins produced by *Bacillus thuringiensis* during sporulation. Diverse families of Cry toxins exhibit toxicity in diverse but different target insects, and are thus used as environment-friendly biological pesticides that can kill the specific insect pests with few side effects for the ecosystem. Genetically modified crops producing Cry toxins have been generated, and 66 million hectares were planted worldwide in 2011 [17]. The mode of action by which Cry toxins kill specific insects is not completely understood. However, toxin-receptor interaction is regarded as an essential step for exerting insecticidal activity, as susceptible insects express Cry toxin receptors such as alkaline phosphatase, aminopeptidase N, cadherin-like protein, or ABC transporter C2 (ABCC2) in the midgut [18]. Upon binding to receptors, Cry toxins form oligomers that generate pores in the cell membrane, leading to cell swelling and osmotic cell lysis. Previous studies have demonstrated that ectopic expression of the *Bombyx mori* receptors cadherin-like protein *BtR175* or *BmABCC2* is sufficient for Cry1A toxins to induce cell death in cultured Sf9 cells, although *BtR175* is less efficient [19]. Since Cry1Aa does not exhibit toxicity against non-target organisms, including *Drosophila melanogaster*, it can be used for conditional cell ablation if the Cry1Aa receptor is selectively expressed. In this report, we established a novel ablation method for rapid and selective induction of cell necrosis achieved by conditional expression of Cry1Aa receptors.

## Results

### Targeted necrosis of Cry1Aa receptor-expressing cells in cultured imaginal discs incubated with Cry1Aa

**Fig. 1:**
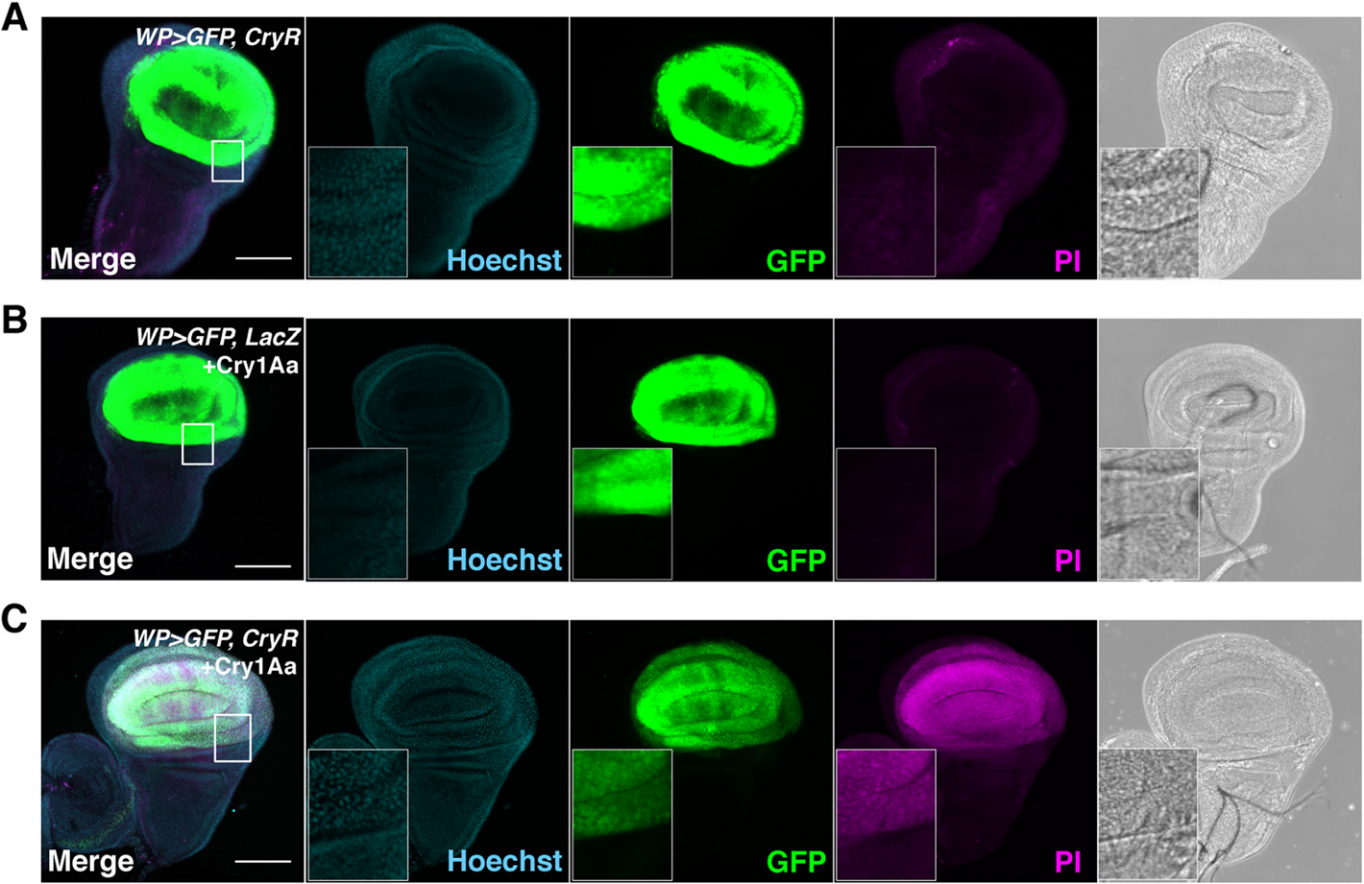
Cry1Aa toxin induces cell necrosis in Cry1Aa toxin receptor (CryR)-overexpressing cells in wing pouch in wing discs. (**a–c**) Propidium iodide (PI) staining of cultured wing discs from third instar larvae expressing *CryR* by *WP-Gal4. WP > GFP*, *CryR* incubated 1 h with 100 nM Cry1Aa showed PI signal (**c**), in contrast to negative controls *WP > GFP*, *LacZ* with 100 nM Cry1Aa (**b**) or *WP > GFP*, *CryR* without Cry1Aa (**a**). Scale bar, 100 μm

To test whether Cry1Aa can induce cell death in cells overexpressing Cry1Aa receptors, we prepared the transgenic flies *UAS-BmABCC2* and *UAS-BtR175*. We overexpressed each receptor in the wing pouch region, which eventually develops into adult wings, using the *WP-Gal4* driver [20]. These flies had no morphological defects in the wing pouch of third instar larvae or in adult wings, suggesting that the ectopic expression of exogenous *Bombyx mori* receptors in these cells was not toxic. We dissected and cultured wing discs from third instar larvae and treated them with Cry1Aa *ex vivo*. We observed propidium iodide (PI)-positive dying cells in the wing pouch region when *BmABCC2*, but not *BtR175*, was overexpressed (Fig. 1a–c, Additional file 1: Figure S1). PI is a nucleic acid stain that acts as a marker for cell death, particularly for necrosis, as it enters the cells only when membrane integrity is disrupted. Since *BmABCC2* is sufficient for Cry1Aa-induced cell death, we designated this receptor as CryR (Cry1Aa toxin receptor) for simplicity. We defined Cry1Aa-induced osmotic cell lysis as a form of necrotic cell death (or accidental cell death) that was not genetically regulated.

**Fig. 2:**
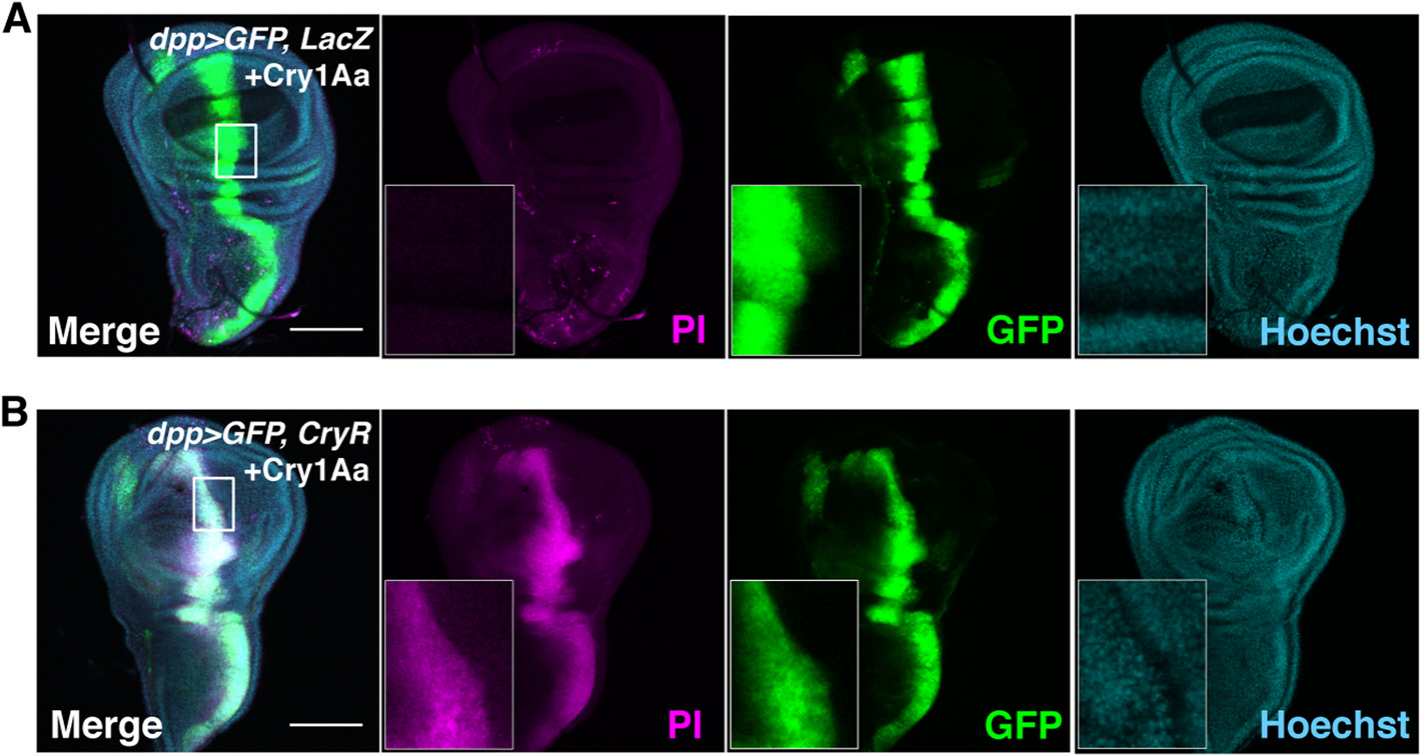
Cry1Aa toxin induces cell necrosis in Cry1Aa toxin receptor (CryR)-overexpressing cells in *dpp > GFP*, *CryR* wing discs. (**a, b**) PI staining of cultured wing discs from third instar larvae expressing *CryR* by *dpp-Gal4.dpp > GFP*, *CryR* incubated 1 h with 100 nM Cry1Aa showed PI signal (**b**), while *dpp > GFP*, *LacZ* incubated with Cry1Aa did not (**a**). Scale bar, 100 μm

**Fig. 3:**
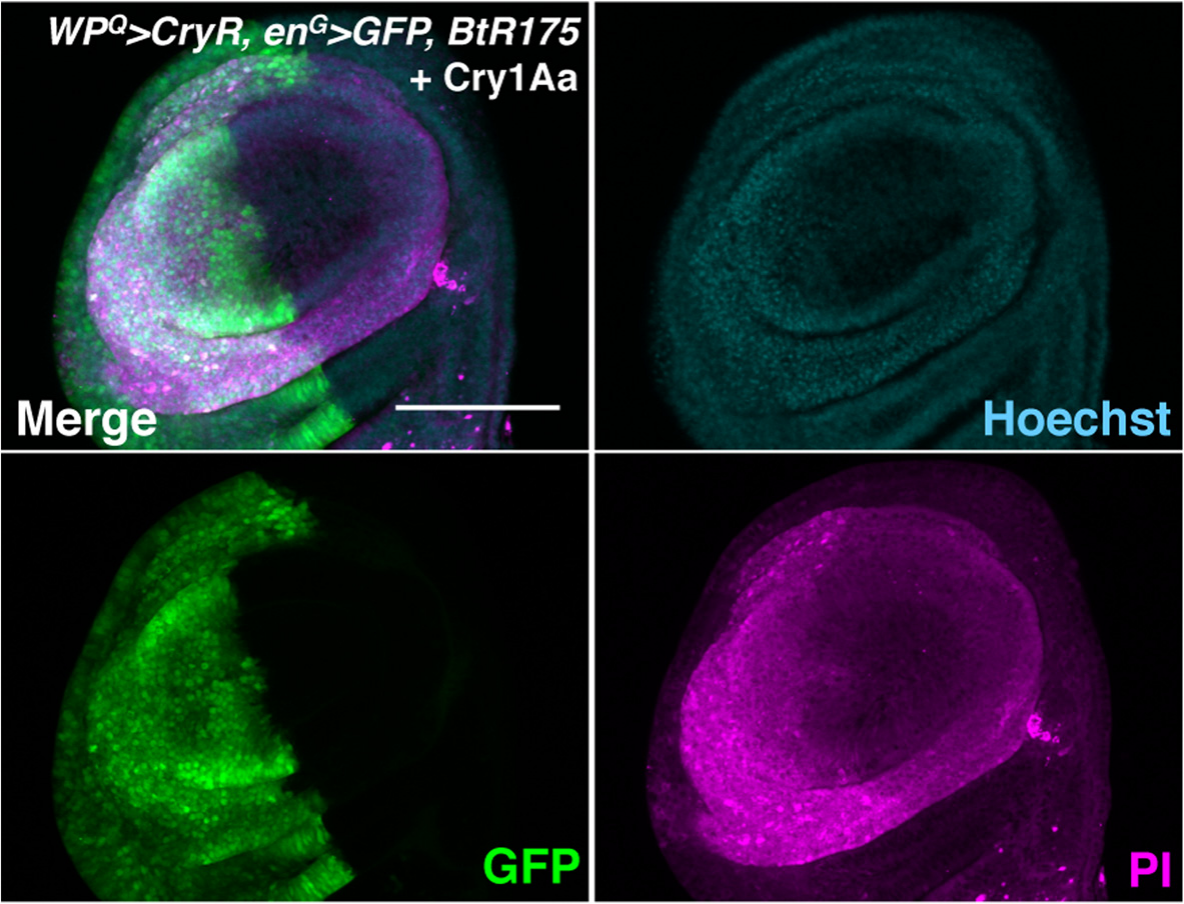
Synergistic effect of simultaneous expression of CryR and BtR175 on Cry1Aa-induced toxicity. Propidium iodide (PI) staining of wing discs from third instar larvae overexpressing *BtR175* by *en-Gal4* and *CryR* by *WP-QF2* incubated for 1 h with 50 nM Cry1Aa. Genotype is *w*; *en-Gal4*, *UAS-GFP/+*; *WP-QF2*, *UAS-BtR175/QUAS-CryR*. PI signals were stronger in the posterior half of the wing pouch where both BtR175 and CryR were expressed. Scale bar, 100 μm

When CryR was driven by *dpp-Gal4*, which expresses at the midline region of wing discs, strong PI signals were observed in CryR-expressing cells when incubated with Cry1Aa (Fig. 2a, b). Simultaneous expression of both receptors further enhanced susceptibility to Cry1Aa-induced cell death (Fig. 3), similar to the synergistic effect observed in Sf9 cells [19]. Serial dilutions of Cry1Aa in the culture medium (concentrations 6.25–100 nM) revealed that 12.5 nM Cry1Aa induced weak but significant PI staining. This became stronger as the toxin concentration was increased; therefore, dose-dependent control of the strength of ablation might be possible (Additional file 1: Figure S2). These data indicate that the Cry1Aa/CryR ablation system can be used to induce conditional cell necrosis in *Drosophila*.

### Rapid and selective cell necrosis by Cry1Aa in cultured imaginal discs and fat body

**Fig. 4:**
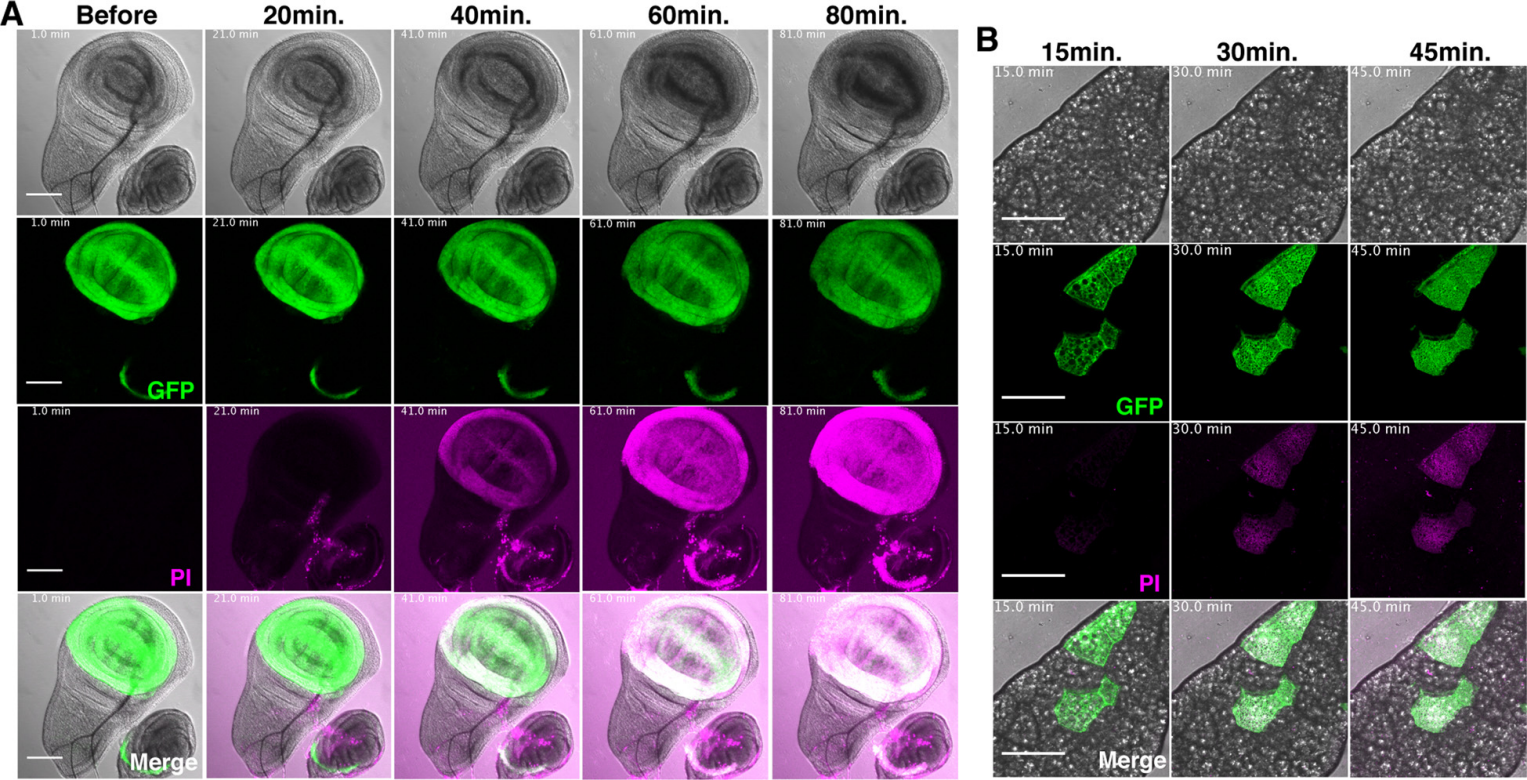
Live imaging of cell necrosis in cultured wing discs and fat body. **a** Time lapse imaging of Cry1Aa-induced cell death in wing discs from third instar larvae. Wing discs of *WP > GFP*, *CryR* were cultured and Cry1Aa was added to the medium at a final concentration of 200 nM. Images were from just before addition of Cry1Aa or after 20, 40, 60, and 80 min incubation. Full movies are supplied online (Additional file 2: Movie S1, Additional file 3: Movie S2 and Additional file 4: Movie S3). Scale bar, 100 μm. **b** Time lapse imaging of Cry1Aa-induced cell death in fat body from third instar larvae. Fat bodies of *hs-flp* ^*122*^, *UAS-mCD8-GFP*; *Actin > y > Gal4*, *UAS-GFP/+*; *UAS-CryR/+* were incubated with 100 nM Cry1Aa. Flip-out clone cells expressing CryR were labeled with GFP. Images were from 15, 30, and 45 min after incubation. Full movies are supplied online (Additional file 5: Movie S4). Scale bar, 100 μm

To investigate the precise time course of Cry1Aa-induced cell necrosis, we performed live imaging analysis of cultured wing discs from *WP > GFP*, *CryR* with 200 nM Cry1Aa (Fig. 4a, Additional files 2, 3 and 4). PI signals were first detected after around 30 min, and increased throughout the 90-minute incubation period. Interestingly, the morphology of the wing pouch region drastically changed just before PI-positive cells were first observed. We observed swelling of GFP-positive cells, which resulted in expansion of the wing pouch region. The *WP-Gal4* driver was also expressed in a small population of leg discs, and these cells also died by cell swelling following Cry1Aa treatment.

Imaginal discs are proliferating tissues that seem to be relatively sensitive to cell death stimuli. Therefore, we investigated whether the Cry1Aa/CryR ablation system was applicable to non-proliferating tissues, such as the fat body, by live imaging analysis (Fig. 4b, Additional file 5). The fat body, a counterpart of mammalian liver and white adipose tissue, is composed of large, postmitotic, polyploid cells. We overexpressed CryR randomly in fat body cells using the flip-out clone technique, which simultaneously labels CryR-positive cells with GFP. When incubated with 100 nM Cry1Aa, CryR-expressing cells became PI-positive 20 min after toxin treatment, without any effect on neighboring CryR-negative cells. This suggests that the Cry1Aa/CryR system is a highly selective method for inducing necrosis with single cell resolution.

### Cry1Aa induced necrosis independently of apoptosis, c-Jun N-terminal kinase (JNK) activation, or autophagy

**Fig. 5:**
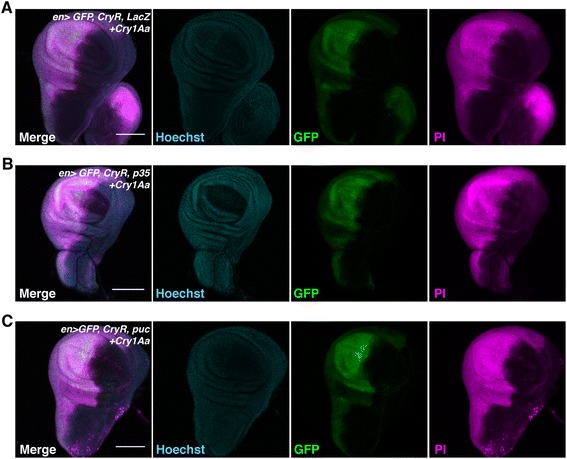
Cry1Aa induces cell death even in cells with inhibited apoptosis or JNK-dependent cell death. (**a–c**) Propidium iodide (PI) staining of cultured wing discs from third instar larvae. *En > GFP*, *CryR* was crossed with *LacZ* (**a**), inhibitor of apoptosis, *p35* (**b**), and JNK inhibitor, *puc* (**c**), and wing discs from F1 progeny were incubated for 1 h with 100 nM Cry1Aa. Scale bar, 100 μm

One of the limitations of conventional cell ablation systems is their dependency on the cell’s ability to activate programmed cell death. In contrast, Cry1Aa directly forms a pore on the plasma membrane, suggesting that cellular context does not affect toxicity. To test this, we overexpressed *p35*, a baculoviral inhibitor of apoptosis that inactivates caspases, and tested whether Cry1Aa could still induce cell death (Fig. 5a, b). As expected, PI signals were not attenuated by p35 overexpression. In addition, Cry1Aa-induced cell death was not blocked by treatment with z-VAD-fmk, a pan-caspase inhibitor (Additional file 1: Figure S3A). We further confirmed that knock down of either *dronc*, an initiator caspase, or of pro-apoptotic RHG genes, did not inhibit cell death (Additional file 1: Figure S3B, C), indicating that apoptosis is not required for the Cry/CryR system.

JNK activation can also induce programmed cell death [21]. For example, Eiger, a tumor necrosis factor superfamily protein in *Drosophila*, kills cells in a JNK-dependent manner, at least partially by a different mechanism from apoptosis. We therefore overexpressed *puckered* (*puc*), which is a negative regulator of JNK. Similar to *p35*, *puc* expression did not affect Cry1Aa-induced cell necrosis (Fig. 5c).

Autophagic cell death is another type of programmed cell death [22, 23]. For example, during metamorphosis in *Drosophila*, degeneration of larval salivary gland or midgut requires autophagic components [24, 25]. When autophagy was inhibited by knock down of *atg1* alone, or together with z-VAD-fmk, we still observed PI-positive dying cells induced by Cry1Aa (Additional file 1: Figure S3D-F). These data demonstrated that Cry1Aa-induced target cell ablation does not require genetic components for cell death.

### Cry1Aa injection induced selective cell necrosis *in vivo*

**Fig. 6:**
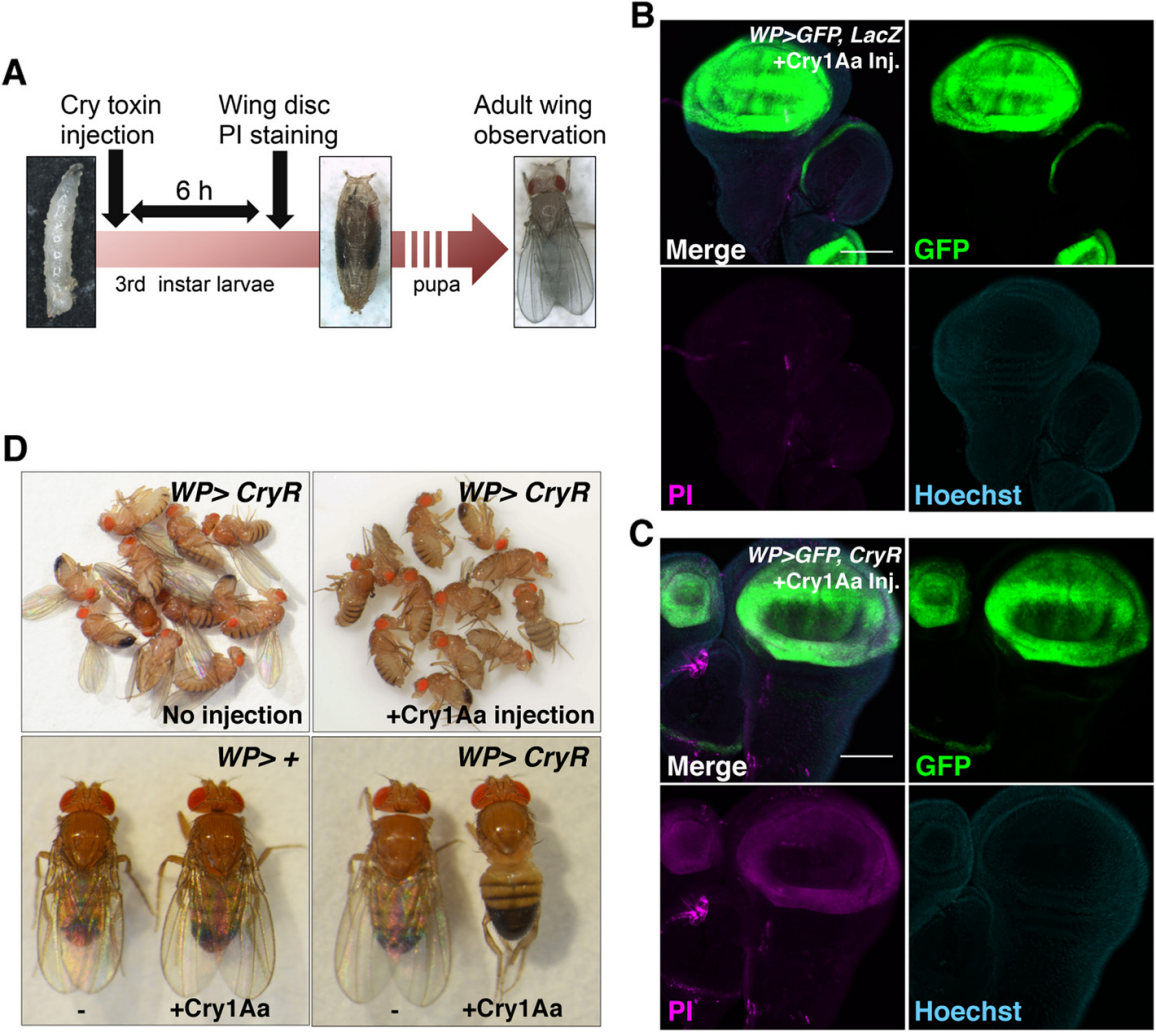
Injection of Cry1Aa for *in vivo* cell ablation. **a** Schematic of an *in vivo* ablation experiment. (**b, c**) Propidium iodide (PI) staining of wing discs from Cry1Aa-injected larvae. All CryR-expressing cells, marked by green fluorescent protein (GFP), were PI-positive (**c**), in contrast to the controls, overexpressing *LacZ* (B). Scale bar, 100 μm. **d** Adult flies injected with Cry1Aa during third instar larvae lost their wings. This phenotype is not observed in the absence of either *CryR* or Cry1Aa injection

To investigate whether the Cry1Aa/CryR system can be used for conditional cell ablation *in vivo*, Cry1Aa was fed to either developing larvae or adult flies through a Cry1Aa-containing diet. However, this failed to kill the animals even though *CryR* was overexpressed ubiquitously (*da >CryR*) or in gut enterocytes (*NP1 >CryR*), probably due to the instability of Cry toxin in the *Drosophila* medium and/or in the digestive tract. Therefore, we injected concentrated Cry1Aa directly into the body cavity of wandering third instar larvae (Fig. 6a). Injection of Cry1Aa into control flies had no apparent effect on viability or development, suggesting that there is little “off-target effect” on flies without exogenous CryR. In contrast, when Cry1Aa was injected into *WP >GFP*, *CryR* larvae, we observed selective cell necrosis in the wing pouch region (Fig. 6b, c). If cells in the wing pouch undergo successful ablation, injected larvae should become adult flies without wings. Strikingly, just a single Cry1Aa injection in third instar larvae resulted in adults without wings (Fig. 6d). This phenotype was only observed in flies expressing CryR and exposed to Cry1Aa, indicating conditional cell ablation by the Cry1Aa/CryR system *in vivo*.

**Fig. 7:**
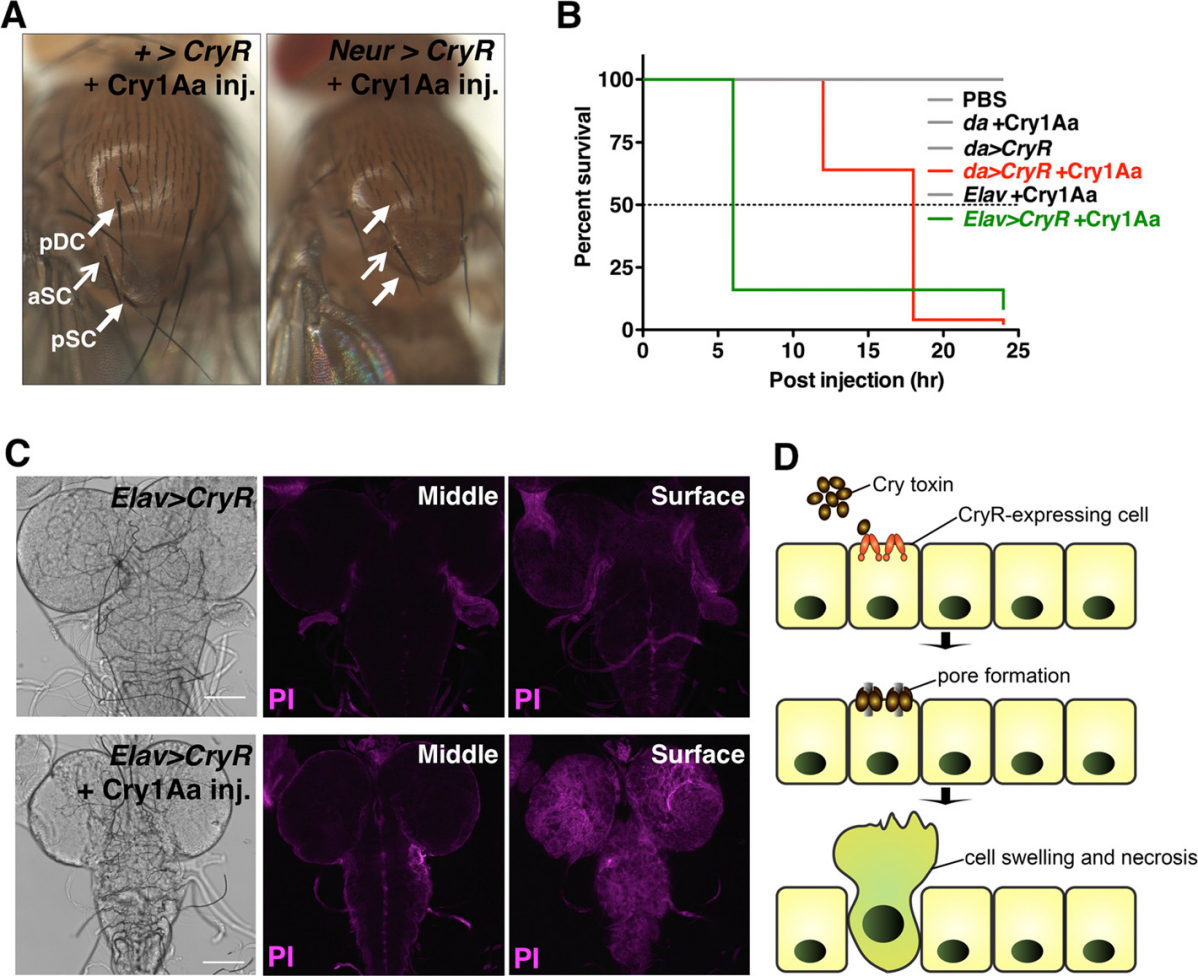
*In vivo* cell ablation of peripheral or central nervous system by Cry1Aa injection. **a** Adult flies injected with Cry1Aa during third instar larval stage lost their bristles. Arrows in the left panel (negative control, without Neur-Gal4) indicate bristle positions (pDC, posterior Dorsocentral; aSC, anterior Scuteller; pSC, posteior Scuteller) and arrows in the right panel (*Neur > CryR*) indicate the presence (aSC) or absence (pDC, pSC) of bristles. **b** Survival curve of male flies with *CryR* expression throughout the whole body (da) or only in neurons (Elav) with and without Cry1Aa; n = 50 for each condition. **c** Confocal images of Propidium iodide (PI) staining of larval brain from *Elav > CryR*, with and without Cry1Aa injection. Brains were dissected 3 h post-injection and then stained with PI. A single focal plane from the middle and surface regions are shown. **d** Schematic view of selective cell ablation by the Cry1Aa/CryR system. Cry1Aa specifically induces cell swelling and necrosis in CryR-expressing cells by pore formation in the plasma membrane

We also tested whether our system is applicable to the developmental study of sensory organs by injecting Cry1Aa toxin into *Neur >CryR* third-instar larvae. *Neur-Gal4* is expressed in sensory organ precursors (SOPs) in wing discs that become adult bristles. Cry1Aa injection during late-third instar larvae (6–12 h before pupal formation) resulted in a loss of bristles from epithelia (Fig. 7a). Although not all bristles are lost, the loss of bristles is probably due to differences in developmental timing as some SOPs such as the anterior scuteller bristle, which arise during later stages of development (0–6 h before pupal formation). Indeed, ablated macrocheates such as posterior scuteller bristle, or posterior drosocentral bristle are generated in early to middle-third larval stage (12–30 h before pupal formation) [26]. This suggested that Cry1Aa kills cells rapidly upon injection into larvae, and that Cry1Aa can be inactivated or removed from the hemolymph within a relatively short period after injection. Therefore, the Cry1Aa/CryR system could be useful for spatiotemporal cell ablation. Furthermore, a lack of melanization in adult epithelia at the original SOP positions implied that dying SOPs are eventually eliminated from the tissue rather than remaining in the epithelial sheet.

We observed a lethal effect of injected Cry1Aa on adult male flies with ubiquitous expression of CryR (Fig. 7b). Almost all CryR-expressing flies died within 18 h of the injection, while all control flies survived. In addition, Cry1Aa injection into *Elav > CryR* where *CryR* was overexpressed in neurons, resulted in an unsteady gait that eventually led to organismal death as early as 6 h post-injection (Additional file 6, Fig. 7b), suggesting that CryR-expressing neurons were damaged by Cry1Aa. To test whether Cry1Aa could permeate across the blood–brain barrier (BBB) and induce cell death in CNS, we performed PI staining upon Cry1Aa injection into larval hemolymphs. The injected brain from *Elav > CryR* larvae were positive for PI staining, but only at the surface of the brain tissue (Fig. 7c). BBB in larval brain is established by surface glia, which are distinct from cortex glia [27, 28]. To further validate that Cry1Aa cross the BBB, we overexpressed *CryR* by cortex glia driver, *Nrv2-Gal4*, and then investigated whether injected Cry1Aa into the hemolymph could induce cell death in these glia. Compared to the control (without Cry1Aa), we observed a large portion of PI-positive cortex glia, although PI-positive cells observed were not limited to GFP-positive cells (Additional file 1: Figure S4). Therefore, we believe that Cry1Aa penetrated the BBB, just as DT did in mice [8], although cells deep inside the tissue were not affected, suggesting that Cry1Aa/CryR system might be applicable for CNS.

## Discussion

Inducing cell death of target cells is increasingly required by both basic biologists, who study the function of specific cells of interest, and clinical scientists seeking selective ablation of unwanted cells such as tumor cells. Because different types of Cry toxins and receptors have different biochemical characteristics, we can select the desired toxin-receptor combinations for each experiment or for each organism. Further, utilizing two receptors can broaden the application. For example, ablation of target cells by expressing two receptors (e.g. CryR and BtR175) simultaneously enhances cell toxicity, as observed in the present study. Moreover, highly specific ablation may be possible using relatively low concentrations of Cry1Aa and two receptors driven by different promoters, which may result in the death of only “merged” cells expressing both receptors.

Recently, Ichikawa et al. [29] developed a method for inducing reactive oxygen species production using a chemical photosensitizer, which was shown to be useful for selective cell ablation in *Drosophila* tissue. This probe (HMDESeR-βGal) is highly conditional, as it is activated by light irradiation only in cells expressing *E. coli* β-galactosidase. However, it is difficult to use this probe for cell ablation in internal tissues, and maintaining tissue in the dark is essential for keeping HMDESeR-βGal latent during the non-ablation period. Therefore, the Cry/CryR system has some advantages, especially for *in vivo* ablation.

The Cry/CryR system provides spatiotemporally regulated induction of cell death. Temporal control of genetic ablation is useful for studying tissue repair or regeneration after tissue injury. Therefore, this system could be used for genetic studies of factors for tissue repair/regeneration. Furthermore, we believe that the Cry/CryR system is probably applicable to selective cell ablation in mammals, including mice, as no obvious toxicity has been observed so far. Conditional expression of CryR by the Cre/LoxP system should permit conditional ablation of mouse cells *in vivo*.

## Conclusions

Cry toxins are widely used for pest control because of their specific insecticidal activity. Our present study demonstrates the possibility of using Cry toxins as a tool for conditional ablation in model organisms like *Drosophila* (Fig. 7d). Since Cry1Aa is a pore-forming toxin that physically damages the target cell membrane, it can induce necrosis in any type or condition of cells, theoretically in any species.

## Methods

### *Drosophila* stocks and genetics

Fly stocks were maintained on a standard diet containing glucose, yeast, and cornmeal at 25 °C and 60 % humidity under 12 h:12 h light:dark conditions. *UAS-Atg1-RNAi* (*HMS02750*), *WP-Gal4* [20], *dpp-Gal4*, *Nrv2-Gal4*, and *en-Gal4* were obtained from the Bloomington *Drosophila* stock center. Clonal expression in the fat body was based on the flip-out technique, as described previously [20]. *WP-QF2* (Kashio et al*.*, in preparation) was constructed from a *QF2* fragment gifted by CJ Potter [30]. *UAS-BtR*, *UAS-CryR*, and *QUAS-CryR* flies were established as described below. *UAS-dronc-RNAi* was established and characterized previously [20]. *UAS-RHG-RNAi* was a gift from CH Chen [31].

### Construction of CryR transgenic flies

To generate the expression constructs *UAS-CryR* and *pQUAST-CryR*, the full length cDNA of CryR/BmABCC2 was amplified by PCR from the pBAC4x-1-EGFP-BmABCC2 vector [19] with primers (5′-CGTgcggccgcATGAATAGTGATGGGAGAG-3′ and 5′-TGAgcggccgcTTTTTCTGTATTTCTACC-3′) or (5′-CGCtctagaATGAATAGTGATGGGAGAGC-3′ and 5′-CGCtctagaTCATTTTTCTGTATTTCTACCAAGATG-3′), and cloned into pUAST or pQUAST vectors using *Not*I or *Xba*I sites, respectively.

To generate the expression construct *UAS-BtR175*, the cDNA of the toxin binding region of BtR175 [32] was obtained by PCR from the pBAC4x-1-EGFP-BtR175-TBR vector [19], with primers (5′-CCGActcgagATGGGAGTTGACGTTCGAAT-3′ and 5′- CCGActcgagTTATGCCAAATTGACAGCTA-3′), and cloned into the pUAST vector using the *Xho*I site.

### Preparation of recombinant active Cry1Aa

Cry1Aa toxin was expressed as a recombinant protein in *E. coli*, and Cry1Aa protoxin was solubilized under highly alkaline conditions (pH 11.0) by NaOH. The solubilized protoxin was activated by trypsin and purified using HPLC, as described previously [33]. The concentration of the purified and activated toxin was measured by densitometry using SDS-PAGE, with bovine serum albumin as a standard.

### PI staining analysis of wing discs *ex vivo*

For PI staining, wing discs were dissected from third instar larvae and incubated with 100 nM (otherwise stated) nM Cry1Aa toxin, 4 μM PI, and 16 μM Hoechst in S2 medium (GIBCO) at 25 °C for 60 min. After extensive washing in S2 medium, the wing discs were fixed with 4 % PFA for 20 min at room temperature and then placed on glass slides. Images were obtained using a Leica SP8 confocal microscope and processed using ImageJ software.

### Live imaging analysis of wing discs and fat body

Wing discs were dissected from late third instar *WP > CryR* larvae in S2 medium and mounted on 35 mm glass-bottomed dishes with 50 μL PBS. After removing the PBS, 500 μL S2 medium was added and time-lapse imaging was performed for 2 h, at 1-min intervals, using a Leica SP5 confocal microscope. Cry1Aa was added to the culture medium at a final concentration of 200 nM between the first and second image acquisitions. For fat body imaging, fat bodies were dissected from late third instar larvae of genotype, *hs-flp*^*122*^, *UAS-mCD8-GFP*; *Actin > y > Gal4*, *UAS-GFP/+*; *UAS-CryR/+*. The fat body was mounted on a glass slide with a small incubation space, with S2 medium containing 100 nM Cry1Aa, and covered by cover glass. After 15-min incubation, time-lapse images were obtained using a Leica SP5 confocal microscope for 30 min at 1-min intervals.

### *In vivo* ablation by Cry1Aa injection

Cry1Aa toxin (13 ng) was directly injected into third instar larvae using the Nanoject II Auto-Nanoliter Injector (Drummond Scientific Company, Broomall, PA, USA). Wing discs were dissected 6 h after injection and stained with PI, or flies were maintained at 25 °C until adult hatching. Larval brains were dissected 3 h post-injection and stained with PI. For adult injection, male flies of *da > CryR or Elav > CryR* were injected with 13 ng Cry1Aa toxin using the Nanoject II Auto-Nanoliter Injector. After injection, the flies were maintained at 25 °C and dead flies were counted every 6 h. For *Elav > CryR* imaging, video was recorded 3 h after the 13-ng Cry1Aa injection.

## Additional files

Additional file 1: Figure S1. CryR, but not BtR175, is sufficient for inducing cell necrosis in *Drosophila* wing discs. Propidium iodide (PI) staining of wing discs from third instar larvae overexpressing BtR175, CryR, or both, with or without 100 nM Cry1Aa incubation for 1 h. Scale bar, 100 μm. **Figure S2.** Dose-dependent response of Cry1Aa-induced cell death in wing discs. Propidium iodide (PI) staining of wing discs from third instar larvae overexpressing CryR. Wing discs were incubated for 1 h with 100, 50, 25, 12.5, or 6.25 nM Cry1Aa. Scale bar, 100 μm. **Figure S3.** Cry1Aa induces cell death in *Drosophila* wing discs in the absence of components for autophagy and apoptosis. Propidium iodide (PI) staining of wing discs from third instar larvae overexpressing CryR in their wing pouch. Discs were incubated with 100 nM Cry1Aa for 1 h. (A) Wing discs were cultured with 100 nM Cry1Aa in the presence of 200 μM pan-caspase inhibitor, z-VAD-fmk. (B, C, D) Knock down apoptotic (B, C) or autophagic (D) components within the wing pouch did not suppress Cry1Aa-induced cell death. Dronc is an initiator caspase. RHG stands for pro-apoptotic genes, Reaper, Hid, and Grim (triple RNAi). (E, F) Simultaneous inhibition of apoptosis by 200 μM z-VAD-fmk and autophagy by *Atg1*-RNAi in the presence (E) or absence (F) of 100 nM Cry1Aa. Scale bar, 100 μm. **Figure S4.** Cry1Aa induces cell death of cortex glia in larval brain *in vivo*. Propidium iodide (PI) staining of brains from third instar larvae that overexpressed CryR by *Nrv2-Gal4*, a cortex glia driver. (A–C) Cry1Aa was injected during the third larval stage and brains were dissected 3 h after injection. (A) No Cry1Aa injection as a negative control. Genotype; *Nrv2>mGFP*, *CryR* Scale bar, 100 μm. (C) High magnification image of Cry1Aa injected brain. Scale bar, 20 μm. Additional file 2: Live imaging of Cry1Aa induced cell death in wing disc from *WP>GFP, CryR*. GFP (green) and PI (magenda) images. Additional file 3: Live imaging of Cry1Aa induced cell death in wing disc from *WP>GFP, CryR*. Merged (GFP and PI) images. Additional file 4: Live imaging of Cry1Aa induced cell death in wing disc from *WP>GFP, CryR*. Bright field images. Additional file 5: Live imaging of Cry1Aa induced cell death in fat body with *CryR*- and *GFP*-overexpressing flip-out clones. PI (magenda) and merged (PI, GFP, and bright field) images. Additional file 6: Movies of *Elav>CryR* adult flies after Cry1Aa injection.

## Abbreviations

ABCC2: ABC transporter C2
BBB: Blood–brain barrier
CryR: Cry1Aa toxin receptor
DT: Diphtheria toxin
JNK: c-Jun N-terminal kinase
PI: Propidium iodide
RHG: Reaper, Hid, Grim
SOP: Sensory organ precursor
UAS: Upstream activation sequence
