# Supplementary material for: Induction of rapid and selective cell necrosis in Drosophila using Bacillus thuringiensis Cry toxin and its silkworm receptor
Source: BMC Biol. 2015 Jul 8;13:48. doi: 10.1186/s12915-015-0160-2 (PMC4495774; doi:10.1186/s12915-015-0160-2)
Supplement: Additional file 1: Figure S1. — CryR, but not BtR175, is sufficient for inducing cell necrosis in Drosophila wing discs. Propidium iodide (PI) staining of wing discs from third instar larvae overexpressing BtR175, CryR, or both, with or without 100 nM Cry1Aa incubation for 1 h. Scale bar, 100 μm. Figure S2. Dose-dependent response of Cry1Aa-induced cell death in wing discs. Propidium iodide (PI) staining of wing discs from third instar larvae overexpressing CryR. Wing discs were incubated for 1 h with 100, 50, 25, 12.5, or 6.25 nM Cry1Aa. Scale bar, 100 μm. Figure S3. Cry1Aa induces cell death in Drosophila wing discs in the absence of components for autophagy and apoptosis. Propidium iodide (PI) staining of wing discs from third instar larvae overexpressing CryR in their wing pouch. Discs were incubated with 100 nM Cry1Aa for 1 h. (A) Wing discs were cultured with 100 nM Cry1Aa in the presence of 200 μM pan-caspase inhibitor, z-VAD-fmk. (B, C, D) Knock down apoptotic (B, C) or autophagic (D) components within the wing pouch did not suppress Cry1Aa-induced cell death. Dronc is an initiator caspase. RHG stands for pro-apoptotic genes, Reaper, Hid, and Grim (triple RNAi). (E, F) Simultaneous inhibition of apoptosis by 200 μM z-VAD-fmk and autophagy by Atg1-RNAi in the presence (E) or absence (F) of 100 nM Cry1Aa. Scale bar, 100 μm. Figure S4. Cry1Aa induces cell death of cortex glia in larval brain in vivo. Propidium iodide (PI) staining of brains from third instar larvae that overexpressed CryR by Nrv2-Gal4, a cortex glia driver. (A–C) Cry1Aa was injected during the third larval stage and brains were dissected 3 h after injection. (A) No Cry1Aa injection as a negative control. Genotype; Nrv2>mGFP, CryR Scale bar, 100 μm. (C) High magnification image of Cry1Aa injected brain. Scale bar, 20 μm. [file 12915_2015_160_MOESM1_ESM.docx]

**
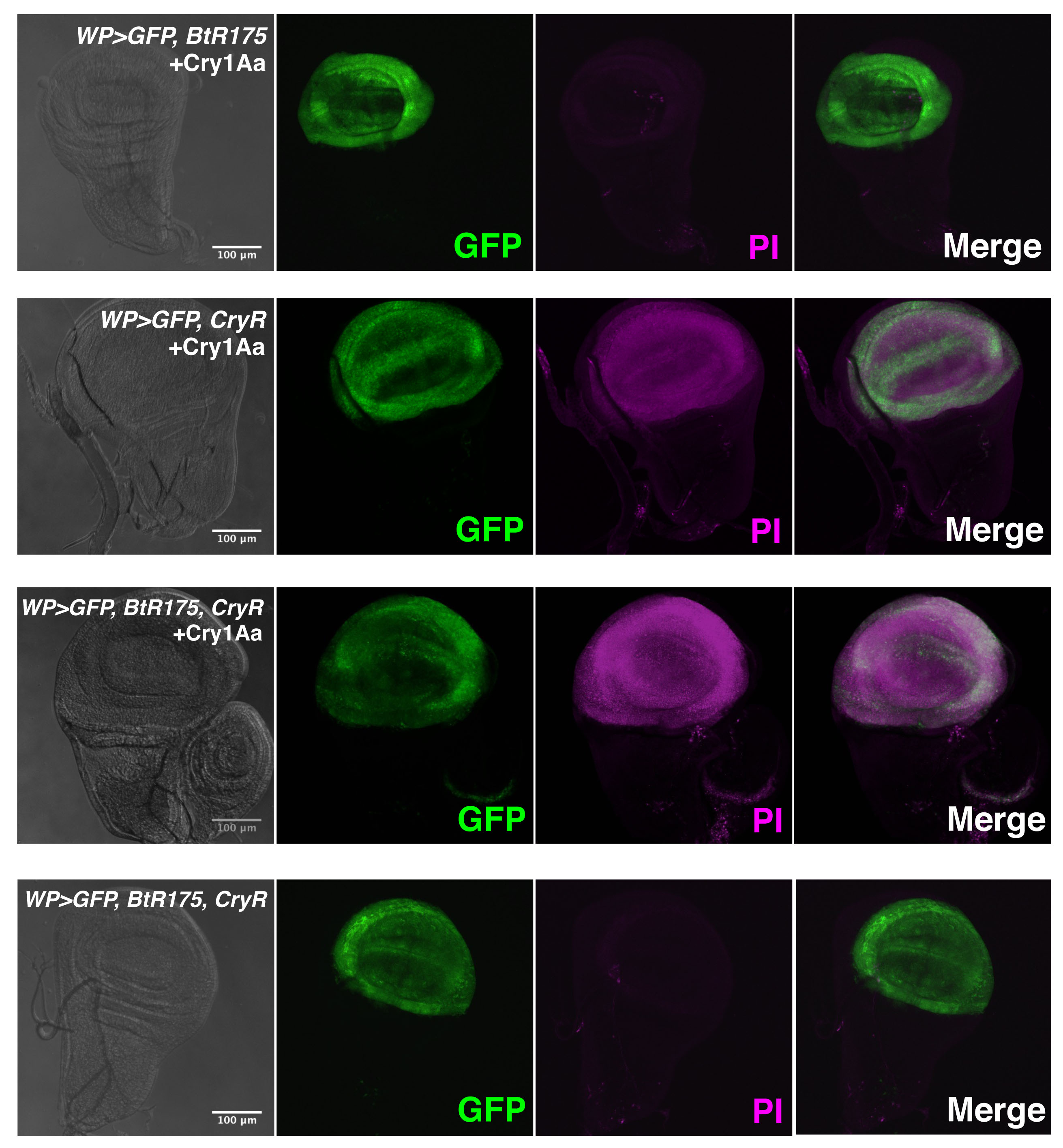
**

**Figure S1. CryR, but not BtR175, is sufficient for inducing cell necrosis in *Drosophila* wing discs.** Propidium iodide (PI) staining of wing discs from third instar larvae overexpressing BtR175, CryR, or both, with or without 100 nM Cry1Aa incubation for one hour. Scale bar, 100 μm.

**
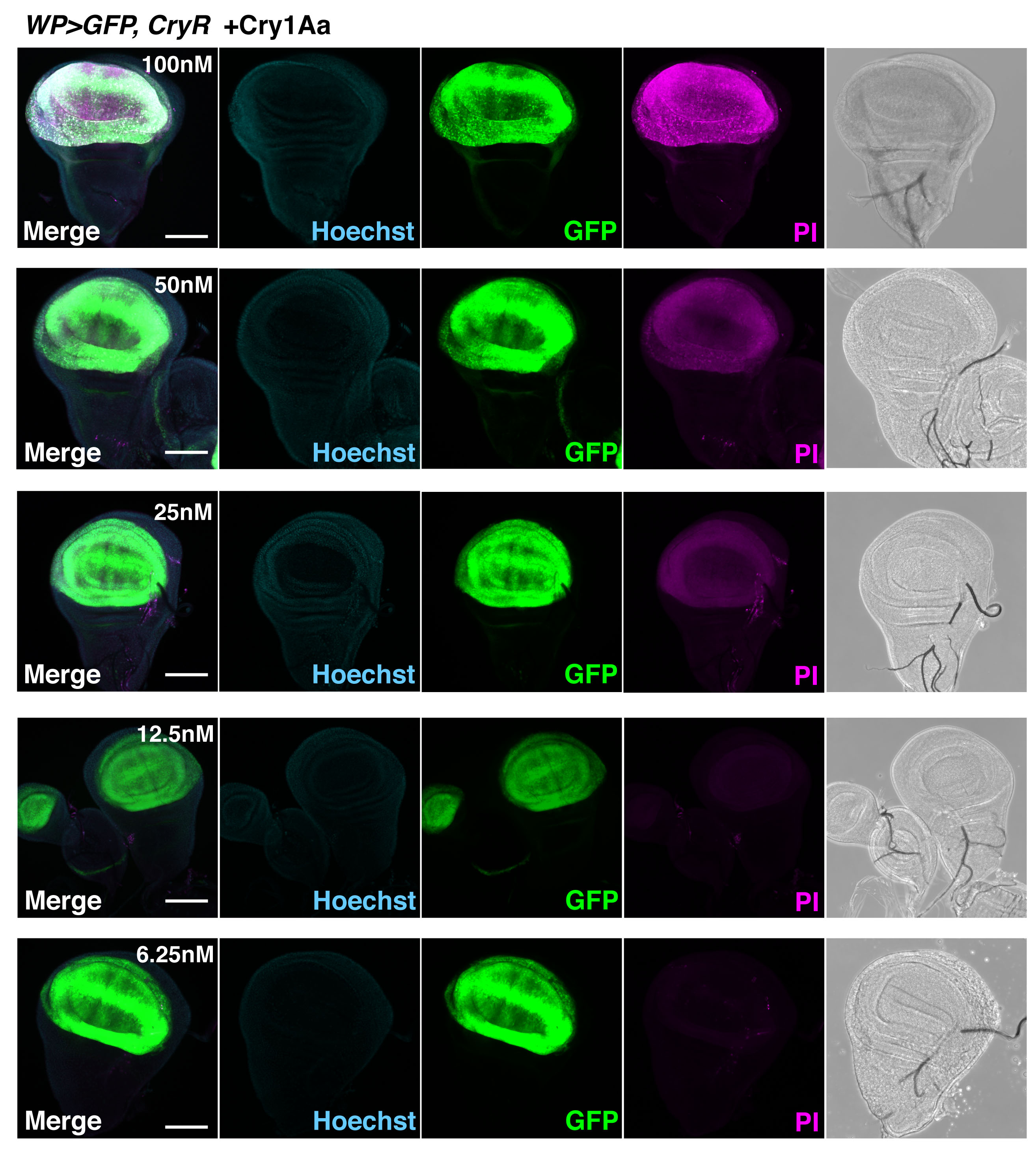
**

**Figure S2. Dose-dependent response of Cry1Aa-induced cell death in wing discs.** Propidium iodide (PI) staining of wing discs from third instar larvae overexpressing CryR. Wing discs were incubated for one hour with 100, 50, 25, 12.5, or 6.25 nM Cry1Aa. Scale bar, 100 μm.


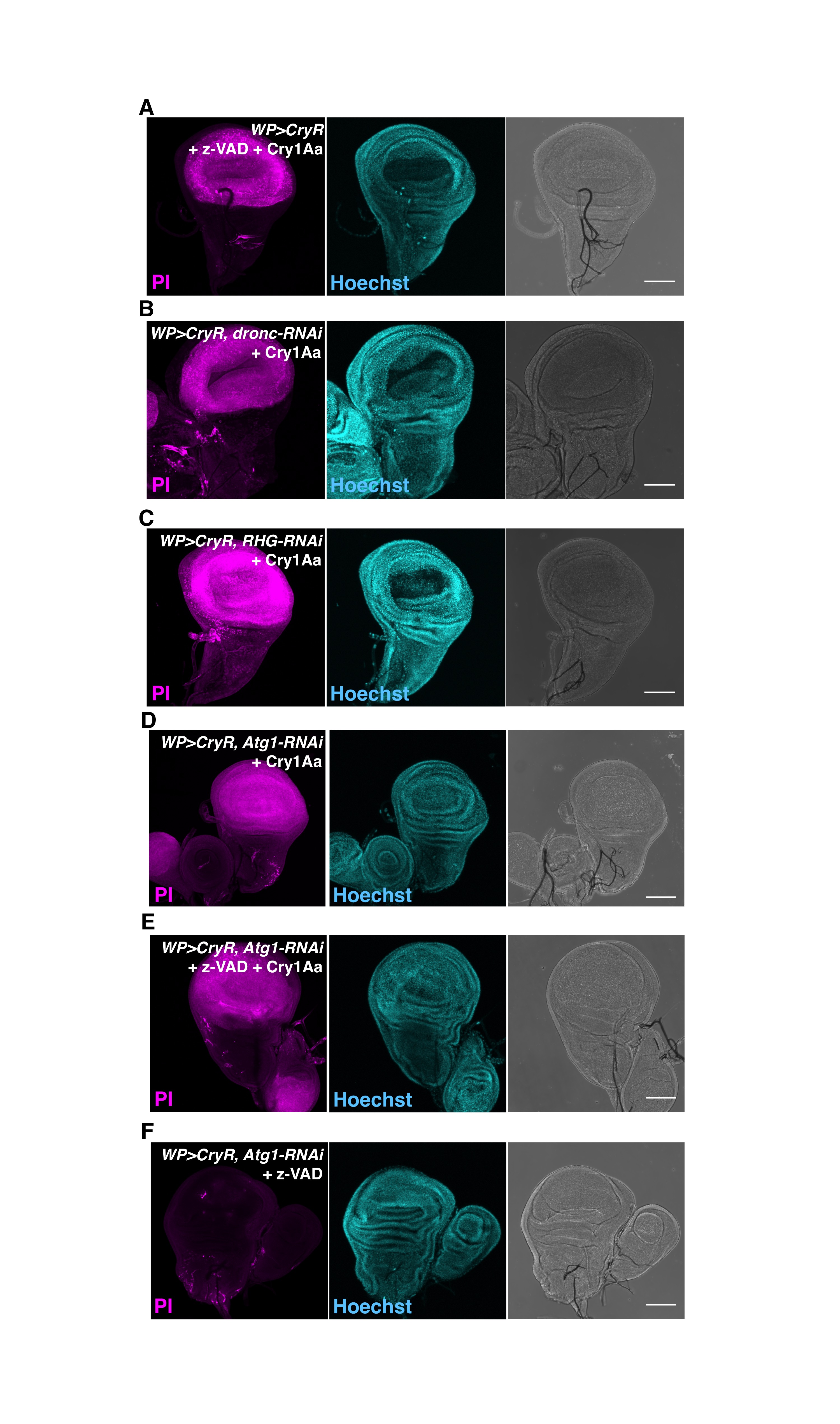


**Figure S3. Cry1Aa induces cell death in *Drosophila* wing discs in the absence of components for autophagy and apoptosis.** Propidium iodide (PI) staining of wing discs from third instar larvae overexpressing CryR in their wing pouch. Discs were incubated with 100 nM Cry1Aa for one hour. (A) Wing discs were cultured with 100 nM Cry1Aa in the presence of 200 μM pan-caspase inhibitor, z-VAD-fmk. (B,C,D) Knock down apoptotic (B,C) or autophagic (D) components within the wing pouch did not suppress Cry1Aa-induced cell death. Dronc is an initiator caspase. RHG stands for pro-apoptotic genes, Reaper, Hid, and Grim (triple RNAi). (E,F) Simultaneous inhibition of apoptosis by 200 μM z-VAD-fmk and autophagy by *Atg1*-RNAi in the presence (E) or absence (F) of 100 nM Cry1Aa. Scale bar, 100 μm.


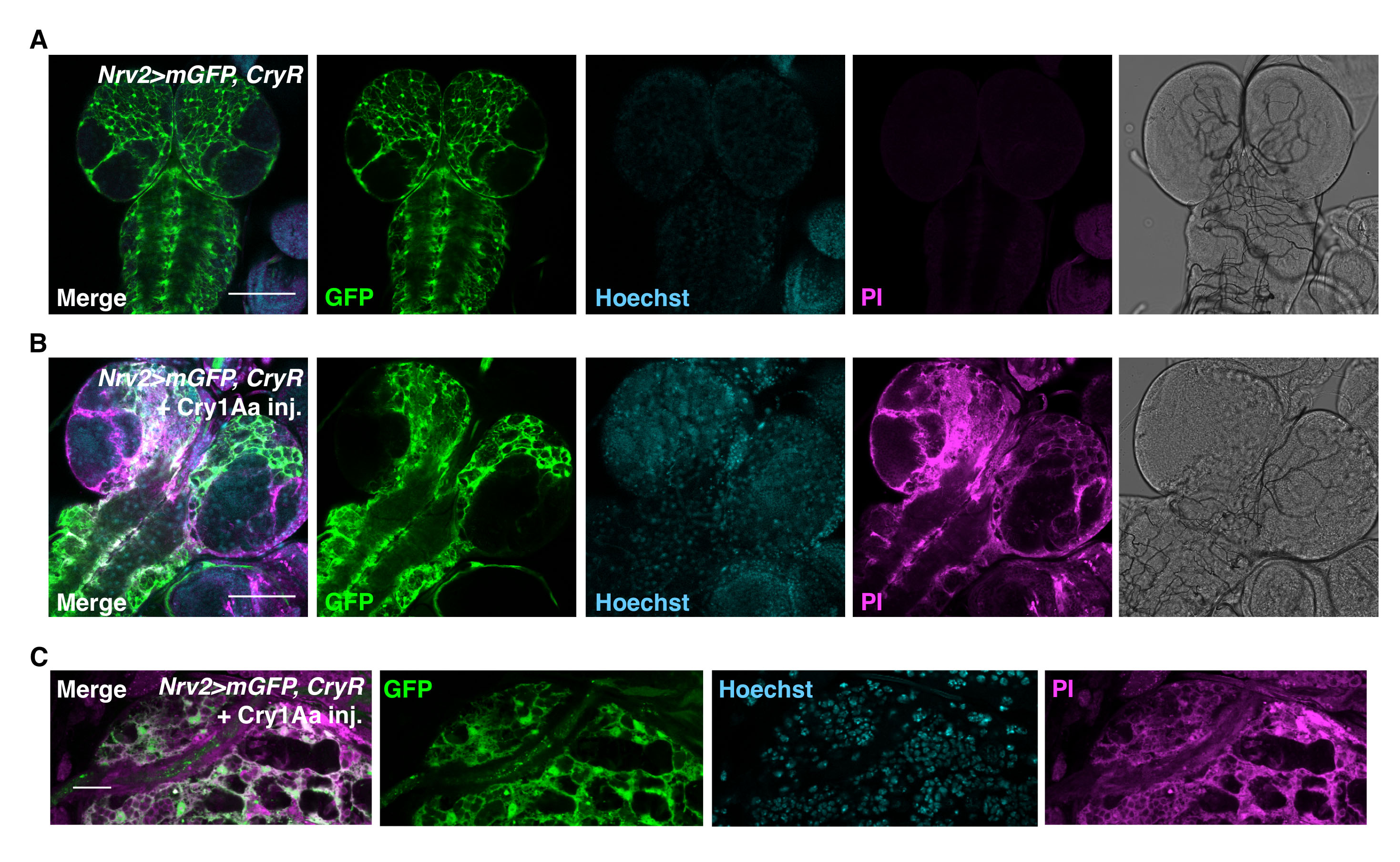


**Figure S4. Cry1Aa induces cell death of cortex glia in larval brain *in vivo*.** Propidium iodide (PI) staining of brains from third instar larvae that overexpressed CryR by *Nrv2-Gal4*, a cortex glia driver. (A-C) Cry1Aa was injected during the third larval stage and brains were dissected three hours after injection. (A) No Cry1Aa injection as a negative control. Genotype; *Nrv2>mGFP, CryR* (A,B) Scale bar, 100 μm. (C) High magnification image of Cry1Aa injected brain. Scale bar, 20 μm.
